# Multi-Omics and Functional Insights into Triterpenoid Biosynthesis Pathways in *Neopicrorhiza scrophulariiflora* (Pennell) D.Y.Hong

**DOI:** 10.3390/plants14101562

**Published:** 2025-05-21

**Authors:** Pinhan Zhou, Juan Wang, Chaohui Li, Lesong Li, Luyuan Duan, Weihao Wang, Xirui Liu, Khadija Tehseen Arshad, Yanli Liang, Yan Zhao

**Affiliations:** 1 Key Laboratory of Medicinal Plant Biology of Yunnan Province, National & Local Joint Engineering Research Center on Germplasms Innovation & Utilization of Chinese Medicinal Materials in Southwest China, Yunnan Agricultural University, Kunming 650201, China; 15663333187@139.com (P.Z.); 15925189272@139.com (J.W.); lichaohui0807@163.com (C.L.); lls15126334094@163.com (L.L.); 18530639128@163.com (L.D.); w771925020@outlook.com (W.W.); lxr1138006812@163.com (X.L.); ktarshadpk@gmail.com (K.T.A.); liangyanlimt@sina.com (Y.L.); 2 College of Agronomy & Biotechnology, Yunnan Agricultural University, Kunming 650201, China; 3 Yunnan Characteristic Plant Extraction Laboratory, Kunming 650106, China

**Keywords:** *Neopicrorhiza scrophulariiflora* (Pennell) D.Y.Hong, triterpenoid, OSCs, bifunctional enzyme, genetic transformation systems

## Abstract

*Neopicrorhiza scrophulariiflora* (Pennell) D.Y.Hong, an endangered perennial herb, is rich in triterpenes, iridoids, and phenolic compounds, which exhibit significant pharmacological effects. However, the molecular mechanisms of triterpenoid biosynthesis in *N. scrophulariiflora* remain unclear. Here, transcriptomic and metabolomic analyses were performed to investigate the triterpene content in different tissues and the expression patterns of key enzyme-encoding genes related to triterpenoid biosynthesis. We functionally characterized eight upstream oxidosqualene cyclases (OSCs) involved in triterpenoid biosynthesis, among which NsOSC2 is a bifunctional enzyme capable of catalyzing the conversion of 2,3-oxidosqualene to *β*-amyrin and *α*-amyrin. Additionally, an efficient regeneration system and a stable genetic transformation system were established for *N. scrophulariiflora*. These findings reveal key genes in triterpenoid biosynthesis, providing a theoretical foundation for the future production of key triterpenoids in *N. scrophulariiflora* through synthetic biology approaches.

## 1. Introduction

*Neopicrorhiza scrophulariiflora* (Pennell) D.Y.Hong, commonly known as *Picrorhiza* genus, is an endangered perennial herb belonging to the Plantaginaceae family. It is primarily found in the Himalayan region, India, and Nepal, with limited distribution in Yunnan, Tibet, and Sichuan Provinces of China, particularly in the Hengduan Mountains [1]. This plant grows at altitudes of 3600 to 4400 m in high mountains, grasslands, rocky areas, or shallow sunny soils. The plant has flat, elliptical leaves with serrated edges [2]. *N. scrophulariiflora* has a unique aroma and bitter taste, with a medicinal history spanning thousands of years. Its medicinal components increase with the plant’s age. The rhizome of *N. scrophulariiflora* is used in medicine, exhibiting hepatoprotective, antioxidant, anti-allergic, anti-asthmatic, etc., effects [3]. Traditionally, the rhizome is boiled in water to treat colds and fever. Recognized as a legitimate Chinese herbal medicine, it is listed in the Pharmacopoeia of China (2020). The pharmaceutical industry incorporates this plant in 22 types of Chinese patent medicines [4]. Additionally, Tibetan pharmaceutical companies use the rhizome of *N. scrophulariiflora* as a raw material in producing Tibetan medicines for hypertension, such as the Twenty-Five Flavors of Yuganzi Pills [5].

Triterpenoids are a class of compounds formed by the combination of triterpenes, which have a basic nucleus composed of six isoprene units (C_5_H_8_) and contain 30 carbon atoms [6]. In traditional Chinese medicines, triterpenoids have gained significant attention due to their potent pharmacological activities. Recent advancements in extraction techniques, such as supercritical fluid extraction, microwave-assisted extraction, and ultrasonic-assisted extraction, have enhanced their isolation. Furthermore, purification methods like macroporous adsorption resin and chromatographic separation have further bolstered the refinement of triterpenoids. They are widely distributed in dicotyledonous plants and are the main active components in many traditional Chinese medicines [7,8], such as ginsenosides in *Panax ginseng* [9], glycyrrhizic acid in *Glycyrrhiza uralensis* [10], loganin in *Siraitia grosvenorii* [11], and saikosaponin (Supplementary Table S1) in *Radix Bupleuri* [12]. Triterpenes are classified based on their C-chain skeleton into linear, bicyclic, tricyclic, tetracyclic, and pentacyclic triterpenes, with tetracyclic and pentacyclic triterpenes [13]. Tetracyclic triterpenes are mainly divided into dammarane, cucurbitane, and cycloartane types, while pentacyclic triterpenes include ursane, oleanane, lupane, etc., types [14].

Oxidosqualene cyclases (OSCs) catalyze the conversion of 2,3-oxidosqualene, which is one of the most complex enzymatic reactions in terpenoid metabolism [15]. The OSC family is diverse, promoting the formation of various triterpenoid compounds in organisms [16]. Individual OSCs can also produce multiple-cyclization products. Based on their ability to produce single or multiple cyclization products [17], OSCs are divided into monofunctional or bifunctional OSCs [18], with monofunctional OSCs being more commonly reported [19]. Bifunctional OSCs have been found in plants such as *Zizyphus joazeiro* [20], *Cucurbita maxima* [21], and *Trichosanthes kirilowii* [22], catalyzing the formation of *β*-amyrin and *α*-amyrin, two pentacyclic triterpenoid skeleton products from 2,3-oxidosqualene. In *Arabidopsis thaliana* and *Pisum sativum*, bifunctional OSCs can simultaneously generate *β*-amyrin, *α*-amyrin, lupeol, and taraxerol, among other pentacyclic triterpenoid derivatives [23] (Supplementary Table S1).

*N. scrophulariiflora* contains various triterpenoids, such as oleanolic and ursolic acids (Supplementary Table S1). Oleanolic acid has antiviral, antibacterial, anti-inflammatory, etc., effects [24]. Ursolic acid exhibits anti-inflammatory, antibacterial, antioxidant, etc., effects [25]. In plants like *G. uralensis* [26], *Polygala tenuifolia* [27], *Aralia elata* [28], and *Ilex cornuta* [29], 2,3-oxidosqualene is catalyzed by *β*-AS to form *β*-amyrin, which is the precursor of oleanane-type saponins. In plants like *Prunella vulgaris* [30], *Malus pumila* [31], and *Centella asiatica* [32], ursane-type saponins are formed by the catalysis of 2,3-oxidosqualene by *α*-AS to form *β*-amyrin. Although OSCs related to triterpenoid biosynthesis have been confirmed in many plants, OSCs related to the formation of triterpenoid skeletons in *N. scrophulariiflora* have not been reported. This study analyzes four tissues of *N. scrophulariiflora*, namely thr flower, leaf, stem, and roots, combining widely targeted metabolomics, transcriptomics, and RT-qPCR technologies to investigate the genes related to the biosynthetic pathway of active triterpenoid compounds in *N. scrophulariiflora*. The identification of genes related to triterpenoid compound synthesis in *N. scrophulariiflora* lays the foundation for enriching the research on this endangered plant and protecting its wild germplasm resources.

## 2. Materials and Methods

### 2.1. Plant Materials

*N. scrophulariiflora* was obtained as the experimental material in this study. The experimental materials were selected from the flowers, stems, leaves, and roots (rhizome and lateral root) of *N. scrophulariiflora* planted in Yulong Autonomous County, Lijiang City, Yunnan Province, China, in May 2023 (100°26′ E, 27°21′ N, 3309.90 m) (Supplementary Figure S1). The plant specimen number is *N. scrophulariiflora*^20230925^, stored at the Kunming Institute of Botany, Chinese Academy of Sciences. Plant samples were collected, and three biological repeats were obtained for transcriptome and metabolome analysis. All samples were immediately frozen in liquid nitrogen after collection and stored at −80 °C until further analysis.

### 2.2. Callus Induction

The *N. scrophulariiflora* seeds were soaked in distilled water for 24 h and then placed in a refrigerator at 4 °C for 24 h for vernalization, and after removing the floating seeds, the seeds were sterilized using HgCl_2_ for 6 min, 75% alcohol for 10 s, and rinsed three times in sterile water. Sterilized seeds were introduced onto MS medium (pH 5.8) supplemented with 30 g/L sucrose, 0.3 mg/L 6-benzylaminopurine (6-BA), 1 mg/L gibberellin 3 (GA3), and 7 g/L agar (seedling induction medium) and incubated under standard culture conditions. After germination, the seedlings were inoculated into MS medium (pH 5.8) contained with 30 g/L sucrose, 0.5 mg/L 1-naphthylacetic acid (NAA), 0.5 mg/L 6-BA, 0.5 mg/L 3-indolebutyric acid (IBA), 0.5 mg/L GA3, 0.5 mg/L chlormequat chloride (CCC), 0.5 mg/L 6-furfurylaminopurine (KT), and 7 g/L agar (shoot induction medium). Subsequently, the seedlings were then cut into 1 cm long stem segments and inoculated into MS medium (pH 5.8) supplemented with 30 g/L sucrose, 0.5 mg/L 6-BA, 1 mg/L NAA, 1 mg/L GA3, 1 mg/L polyvinylpyrrolidone (PVP), and 7 g/L agar (callus induction medium). Once the buds were induced, they were inoculated onto MS medium (pH 5.8) accompanied by 30 g/L sucrose, 0.5 mg/L NAA, 0.5 mg/L 6-BA, 0.5 mg/L IBA, 0.5 mg/L GA3, 0.5 mg/L CCC, 0.5 mg/L KT, and 7 g/L agar (rooting induction medium) to promote root development and complete plant formation.

### 2.3. Metabolite Profiling Using UPLC-MS/MS

The samples (100 mg) were grounded with liquid nitrogen and resuspended in pre-chilled 80% methanol and 0.1% formic acid by well vortexing. The samples were incubated on ice for 5 min and then centrifuged at 15,000× *g*, 4 °C for 20 min. Supernatant was diluted to a final concentration containing 53% methanol by LC-MS-grade water. Data acquisition was conducted with ultra-performance liquid chromatography (UPLC) and a tandem mass spectrometer (QTRAP^®^ 6500+, SCIEX, Canada, Suzhou, China). The liquid phase and mass spectrometry conditions were determined according to previously reported methods.

A predetermined multiple reaction monitoring (MRM) technique was used to analyze experimental samples by Guangzhou Kidio Biotechnology Co., Ltd. (Guangzhou, China). A qualitative and quantitative analysis of metabolites was carried out using SCIEX OSV 1.4 software (Supplementary Table S2).

### 2.4. RNA-Seq Analysis

The mRNA was enriched using mRNA Capture Beads, purified, and then fragmented using high temperature; the first strand of cDNA was synthesized using the fragmented mRNA as a template in a reverse-transcription mixing system. When the second strand of cDNA was synthesized, end repairs were performed, and an A-tail was added. Then, the junctions were connected and the target fragments were purified using Hieff NGS^®^DNA Selection Beads. The library was constructed and sequenced on an Illumina Novaseq X Plus platform (Guangzhou, China), and transcriptome data were obtained (Supplementary Table S3).

The expression level of the transcript was determined by the FPKM value (fragments per kilobase of transcript per million mapped reads). Differentially expressed genes (DEGs) were identified using *p* < 0.05 and |log_2_FoldChange| > 2. To ensure the accuracy of the RNA-seq data, four DEGs were selected for the RT-qPCR assay. The expression levels of different genes and samples were analyzed using the TBtools (v2.210) software package for all comparison groups. The principal component analysis (PCA) was performed to determine the extent of metabolic differences between samples within groups. GO (gene ontology) function and KEGG pathway enrichment were determined by hypergeometric distribution tests, using the Omicsmart online platform (https://www.omicsmart.com, accessed on 13 April 2024). The sequencing data of this study were submitted to the National Center for Biological Information (CNCB https://www.cncb.ac.cn/, accessed on July 2024), China, with the BioProject accession (PRJCA027400).

### 2.5. Phylogenetic Analysis

The OSC protein sequences from *A. thaliana* were downloaded from the NCBI protein database (Supplementary Table S4). Based on the results of PfamScan (version 1.6), *OSC* genes were selected based on the PF13243 annotated sequence. Multiple sequence alignment was performed using MAFFT v7.505, and a phylogenetic tree was constructed by bootstrapping 1000 times with the maximum likelihood method (ML). It was then beautified and visualized using online software, iTOL.

### 2.6. Vector Construction

*Agrobacterium tumefaciens* strain GV3101, pYES2 yeast expression vector, and plant overexpression vector pCAMBIA1300-GFP were used in this study. The *Saccharomyces cerevisiae* strain GIL77 lacked lanosterol, and it was also used in this study. The complete coding sequence of the *NsOSC2* gene (2279 bp) was cloned from *N. scrophulariiflora*. The target fragment was subcloned into the pYES2 yeast expression vector using the primers (Supplementary Table S5), and then the recombinant plasmid was transformed into *S. cerevisiae* GIL77 strain for functional analysis by the LiAc/ss-DNA /PEG method, as well as the target fragment was subcloned into the pCAMBIA1300-GFP vector between the *Xba*I and *Kpn*I restriction sites to generate the pCAMBIA-1300-*NsOSC2*-GFP (OE-*NsOSC2*) construct, and the plasmid was then introduced into *A. tumefaciens* strain GV3101 and transformed in cultured *N. scrophulariiflora* tissues.

### 2.7. Characterization Analysis of NsOSC2 in Yeast

The cloned sequence should be recombinantly inserted into the yeast expression vector pYES2. LiAc/SS-DNA/PEG was used to transform the recombinant plasmid lanosterol-deficient *S. cerevisiae* GIL77. The empty vector pYES2 was used as a control and cultured in SC-Ura liquid medium containing 2% glucose, 20 µg/mL ergosterol, 13 µg/mL hemin chloride, and 5 µg/mL tween 80 at 30 °C and 200 rpm for 2 d. Subsequently, the culture was incubated in SC-Ura liquid medium without ergosterol (supplemented with 2% galactose instead of glucose) for 3 d for yeast expression experiments. Cells are collected by centrifugation, incubated with phosphate buffer solution for 24 h, and then incubated with 20% KOH-ethanol aqueous solution for 3 min at 100 °C, and the active ingredients are extracted with ethyl acetate. After evaporation, we incubated with trimethylsilane at 60 °C for 30 min, and we analyzed yeast expression products using gas chromatography–mass spectrometry (GC-MS). As a product-processing method for GC-MS detection, the extract was vacuum-concentrated at 45 °C and resuspended with 10 ng/μL coprostanol (internal standard) in 200 μL trimethylsilyl cyanide, derivatization at 65 °C, and incubated for 30 min. The transfer line was heated to 250 °C, and the temperature of the ion source was set to 250 °C.

Column temperature was programmed at 80 °C for 2 min and then ramped to 290 °C at 20 °C/min with a 30 min hold. The carrier gas was helium, with a flow rate of 1.2 mL/min. The samples were injected at a ratio of 10:1 with a 1 μL sample volume.

### 2.8. Stable Transformation of N. scrophulariiflora

Recombinant plasmid pCAMBIA-1300-*NsOSC2*-GFP was electroporated into *A. tumefaciens* GV3101 for the stable transformation of *N. scrophulariiflora.* The cultured healing tissues of *N. scrophulariiflora* were immersed in resuspended *A. tumefaciens* solution for 8 min. They were then placed on sterile filter paper to dry, washed twice with sterile water, and dried. MS solid medium supplemented with 1 mg/mL acetosyringone (AS) was used for dark culture for 2 d. They were then transferred to MS solid medium containing 100 mg/L kanamycin for bud induction. Following a period of 10 d of incubation, positive healing wounds were identified.

### 2.9. RT-qPCR Analysis

Flower, leaf, stem, roots, and callus from *N. scrophulariiflora* were ground using liquid nitrogen. Total RNA was extracted with TRIzol reagent. First-strand complementary DNA (cDNA) was synthesized using the TransStart OneStep gDNA Removal and cDNA Synthesis Super Mix kit (TransGen Biotech, TransGen Biotech Co, Ltd., Beijing, China) and Oligo (dT)18 primer in a total volume of 20 μL.

We made a 20 μL reaction mixture using 1 µL cDNA, 8.2 µL distilled water, 10 µL of SYBR qRFPCR Master Mix, and gene-specific primers. The PCR program was performed using the Step One Plus Real-Time PCR System (Applied Biosystems, Waltham, MA, USA), which concluded 1 cycle (95 °C, 30 s), 40 cycles (95 °C, 10 s; 58 °C, 20 s), 1 cycle (95 °C, 15 s), 1 cycle (60 °C, 1 min) and 1 cycle (95 °C, 15 s). Specific primers were designed through the online Primer3 Web version 4.1.0, and four key OSCs involved in triterpenoid biosynthesis were selected for RT-qPCR. Actin was selected as the internal reference gene, and the relative expression levels of candidate genes were calculated using the 2^−ΔΔCt^ method. The primers used for RT-qPCR are included in Supplementary Table S4.

### 2.10. Statistical Analysis

A single-factor experimental design was used in the experiment. Three biological replicates were used for each treatment. GraphPad Prism 9.50 was used for the statistical analysis, and the analysis was performed through a one-way ANOVA. Data and error bars indicate the mean ± standard deviation (SD) of three independent biological replicates.

## 3. Results

### 3.1. The Metabolome Profiling in N. scrophulariiflora

**Figure 1:**
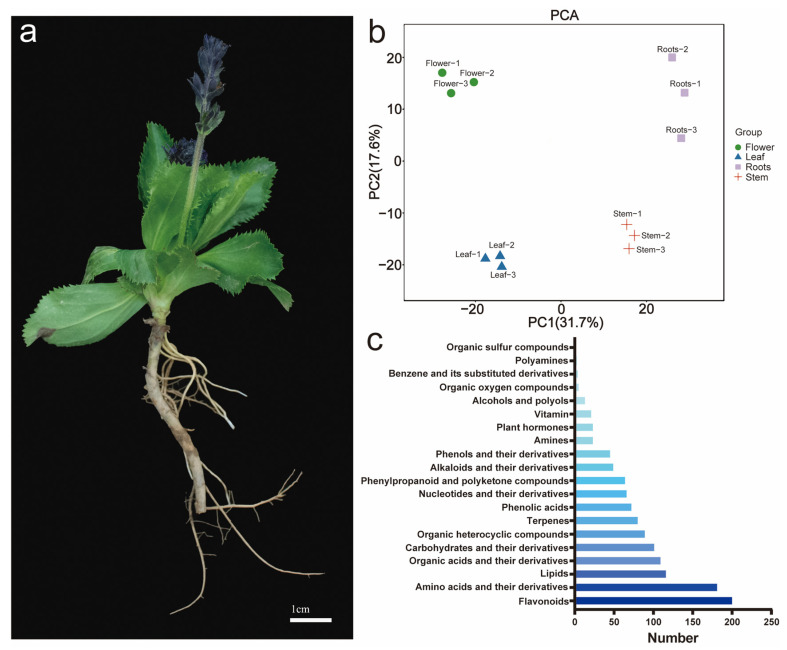
Analysis of metabolites from different tissues in *N. scrophulariiflora*. (**a**) Different tissues of *N. scrophulariiflora* listed from top to bottom: flower, stem, leaf, and roots (including rhizome and lateral root). (**b**) PCA analysis of metabolome data. (**c**) Total metabolite classification, with each row representing a different category.

To investigate the metabolites of the flower, leaf, stem, and roots of *N. scrophulariiflora*, widely targeted metabolomics was established (Figure 1a). UPLC-MS/MS technology was utilized to establish the target metabolism database, and PCA analysis showed significant differences among tissues, indicating that the metabolites of the various tissues differed significantly (Figure 1b).

We compared the similarities and differences between metabolites at *p* < 0.05 and |log_2_FoldChange| > 2, and nine metabolites co-existed in the samples (Supplementary Figure S2). Among the metabolites, 732 were identified by POS (positive-ion mode) and 550 by NEG (negative-ion mode) (Supplementary Table S6), and 214 and 247 were annotated by the KEGG pathway. It contained 201 flavonoids, 182 amino acids and their derivatives, 117 lipids, 110 organic acids and their derivatives, 102 sugars, 90 organic heterocyclic compounds, 82 terpenoids, 73 phenolic acids, 67 nucleotides, 65 phenylpropanoids and polyketides, etc. (Figure 1c).

A further assessment of metabolic differences between tissues was conducted using differentially accumulated metabolites (DAMs). A total of 103 DAMs were identified in the root vs. flower, 79 in the root vs. leaf, 102 in the root vs. stem, 104 in the stem vs. flower, 93 in the stem vs. leaf, and 72 in the leaf vs. flower (Supplementary Figure S3). The Venn plots revealed nine metabolites across the six groups, indicating highly differentiated metabolites among the four tissues. Furthermore, metabolites were the most abundant in the leaf among the four tissues studied, followed by root, flower, and stem. Our analysis of DAMs revealed 1282 metabolites that were annotated into KEGG, and they were predominantly involved in secondary metabolite biosynthesis (Supplementary Figure S4). As terpenoids are active components in the secondary metabolites of *N. scopulariiflora,* further analysis of the terpenoid metabolites revealed that *N. scrophulariiflora* contains 30 triterpenes, 15 sesquiterpenes, 12 cycloenol ether terpenes, 11 monoterpenes, 11 diterpenes, and 2 terpenes, with triterpenes being most abundant in the roots (Supplementary Figure S5).

### 3.2. Identification of Differentially Expressed Genes (DEGs)

**Figure 2:**
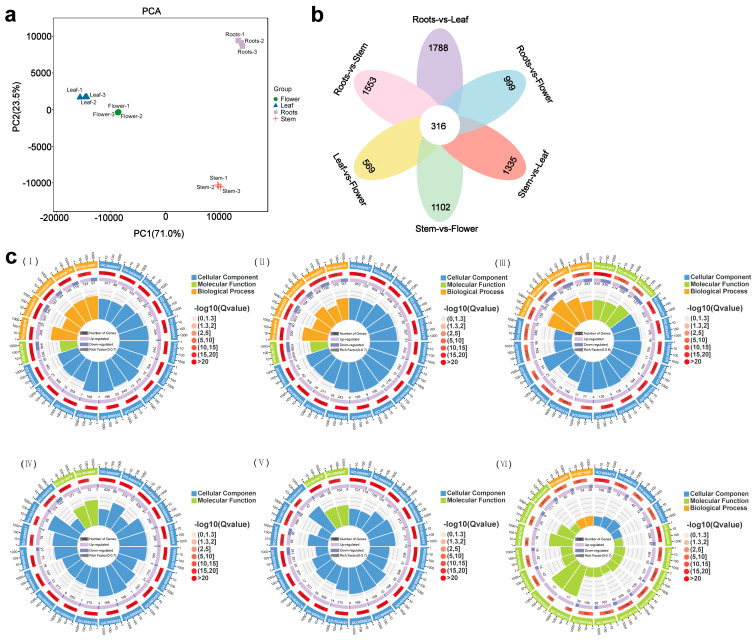
Analysis of DEGs among various tissues of *N. scrophulariiflora*. (**a**) PCA analysis of the RNA-seq data. (**b**) Identification of the DEGs across the six groups analyzed in the RNA-seq. The Venn dia-gram illustrates the results for comparisons between roots vs. leaf, roots vs. flower, stem vs. leaf, stem vs. flower, leaf vs. flower, and roots vs. stem. (**c**) GO enrichment analysis of DEGs. (I) Roots vs. flower, (II) roots vs. leaf, (III) roots vs. stem, (IV) stem vs. leaf, (V) stem vs. flower, and (VI) leaf vs. flower. Different colors represent various pathway classifications, while varying heights of the bars indicate the number of genes enriched for each pathway. Shades of red reflect the enrichment levels of DEGs within the GO term.

To determine the gene expression patterns in different tissues, we conducted RNA-seq on *N. scrophulariiflora* and performed PCA analysis on all samples. The results showed that the same tissues of plants had a high biological repeatability (Figure 2a). Following this, 316 DEGs were identified at *p* < 0.05 and |log_2_FoldChange| > 2 (Figure 2b).

A total of 22,144 DEGs were identified as being between the root vs. flower, 23,780 DEGs between the root vs. leaf, 19,435 DEGs between the root vs. stem, 1714 DEGs between stem vs. flower, 13,890 DEGs between stem vs. leaf, and 7935 DEGs between leaf vs. flower (Supplementary Figure S6). The DEGs in stem, flower, and leaf were primarily upregulated compared with the root. As a result of KEGG enrichment, upregulated DEGs showed enrichment in the terpenoid backbone biosynthesis pathway, whereas downregulated genes did not show any enrichment (Supplementary Figure S7).

Based on GO enrichment analysis, these DEGs were enriched in biological processes (BPs), molecular functions (MFs), and cellular components (CCs) (Figure 2c). At the same time, DEGs enriched in KEGG pathways include secondary-metabolite synthesis (Supplementary Figure S8), which is consistent with what was observed in DAMs.

### 3.3. Phylogenetic and Expression Analysis Related to Triterpenoid Biosynthesis

**Figure 3:**
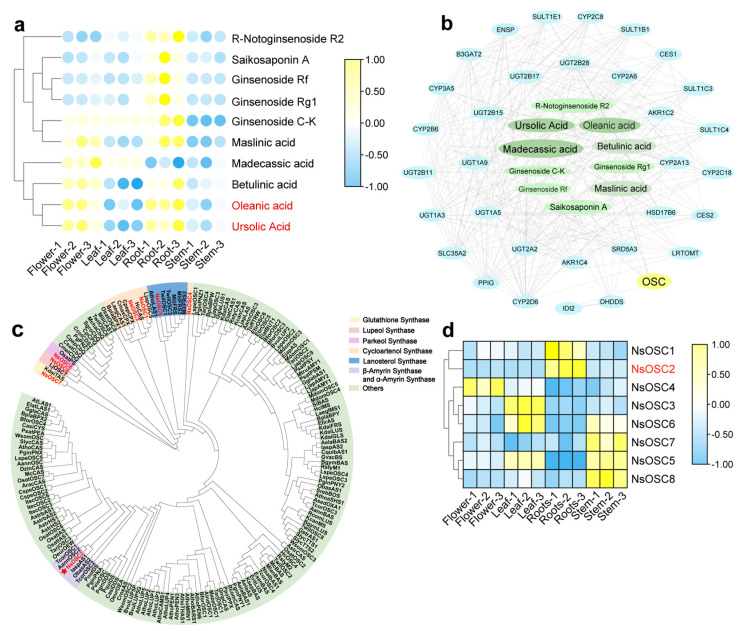
System analysis of OSCs. (**a**) Heatmap of metabolites of pentacyclic triterpenes: The heat map illustrates the Z-score calculated from the relative content of metabolites across different tissues, with colors ranging from blue to yellow (low to high). Key metabolites are highlighted in red. (**b**) co-expression network analysis of candidate genes and pentacyclic triterpenoid metabolites: The co-expression network analysis displays interactions between candidate genes and pentacyclic triterpenoid metabolites. Node colors represent different genes or metabolite classes, while darker colors indicate stronger connections. (**c**) Phylogenetic tree of *OSCs* in *N. scrophulariiflora.* A phylogenetic tree was constructed based on the eight *OSC*s screened in *N. scrophulariiflora* and other species (Supplementary Table S3). Different colors in the tree denote various classifications. The asterisk indicates the genes for subsequent functional verification. (**d**) Heatmap of target genes: The heatmap presents *Z*-scores calculated from the relative content of genes across different tissues, with colors ranging from blue to yellow (low to high). Key genes are highlighted in red.

To determine triterpenoid biosynthesis pathways, we analyzed DAMs and DEGs. Further investigation of triterpenoid accumulation, therefore, focused on 2,3-oxidosqualene cyclases (OSCs), the key enzymes responsible for creating the triterpenoid skeleton. OSCs catalyzed the formation of some triterpene skeletons in metabolome, where pentacyclic triterpenoids were found to be present at higher levels in roots (Supplementary Figure S9). To perform a heatmap of the metabolites, nine metabolites from the three compounds were selected, each of which had a pentacyclic triterpene skeleton. The highest levels of triterpene skeletons in roots were found in ursolic acid and oleanolic acid, followed by R-notoginsenoside R2 and ginsenoside C-K (Figure 3a).

Further screening of candidate genes involved in *N. scrophulariiflora* biosynthesis was carried out by analyzing the correlation between 10 OSC synthesis-related DAMs (Supplementary Figure S10) and 37 *OSC* synthesis-related DEGs. Nine DAMs were significantly correlated with 37 structural DEGs. Oleanolic acid, madecassic acid, and ursolic acid have the best connectivity among them, followed by betulinic acid and maslinic acid. One of the tightly connected DEGs is OSCs, suggesting that it may be a key enzyme in the synthesis pathway of *N. scrophulariiflora* (Figure 3b).

To identify the OSC genes family in *N. scrophulariiflora,* eight *OSC* genes were identified and named *NsOSC1*-*NsOSC8.* The phylogenetic relationship between *OSC* genes in *N. scrophulariiflora* was assessed by combining the screened *NsOSC* genes with previously reported functional OSCs (Figure 3c).

Phylogenetic analysis shows that NsOSC1, NsOSC3, and NsOSC4 have the closest homology with cycloartenol synthase; NsOSC2 has close homology with *β*-amyrin synthase and *α*-amyrin synthase; NsOSC5 has the closest homology with lanosterol synthase; NsOSC6 has close homology with lupeol synthases; NsOSC7 has the closest homology with glutathione synthases; and NsOSC8 has close homology to parkeol synthase. Therefore, we speculate that NsOSC1, NsOSC3, and NsOSC4 are involved in the biosynthesis of cycloartenol synthase; NsOSC2 is involved in the biosynthesis of *β*-amyrin synthase and *α*-amyrin synthase; NsOSC5 is involved in the biosynthesis of lanosterol synthase; NsOSC6 and NsOSC8 are involved in the biosynthesis of lupeol synthases; and NsOSC7 is involved in the biosynthesis of glutinol synthases.

Thus, it can be speculated that the biosynthesis of triterpenoids in *N. scrophulariiflora* is controlled by multiple DEGs, of which *OSC* genes play a key role. Among the eight *OSC*s obtained, *NsOSC4* had the highest expression level in the flower; *NsOSC3* and *NsOSC5* had the highest expression level in the leaf; *NsOSC2* had the highest expression level in the roots; and *NsOSC5* and *NsOSC8* had the highest expression level in the stem (Figure 3d).

### 3.4. Verification of Genes Related to Triterpenoid Biosynthesis Using RT-qPCR

**Figure 4:**
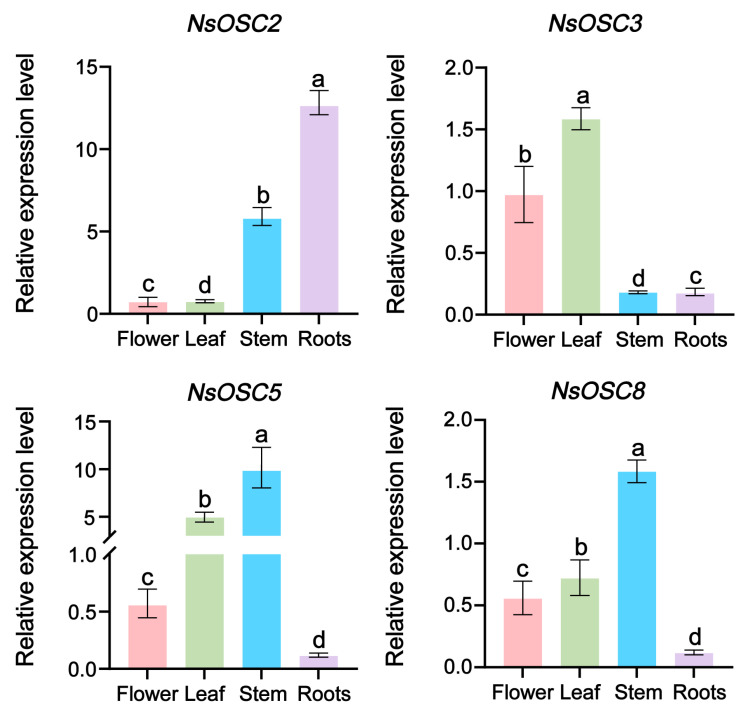
RT-qPCR validation of key enzyme genes involved in triterpenoid synthesis. The y-axis shows the relative expression level, and the x-axis represents different tissues. The colors denote different plant tissues: pink represents the flower, green represents the leaf, blue represents the stem, and purple represents the roots. Note: The mean was ±SD; three independent biological replicates and different letters on the square columns indicate significant differences between treatments, as determined by one-way ANOVA analysis.

*OSC* genes play a crucial role in triterpenoid biosynthesis. Therefore, the OSC genes family of *N. scrophulariiflora* was further screened, and four genes were identified, namely *NsOSC2*, *NsOSC3*, *NsOSC5*, and *NsOSC8*. The SnapGene software package was used to design primers for gene cloning (Supplementary Table S6), and the four genes mentioned above were successfully cloned and then used to develop RT-qPCR primers for experiments that identified and amplified the four genes involved in triterpenoids biosynthesis from *N. scophulariiflora* (Supplementary Table S7). According to the results, *NsOSC2* has the highest expression level in the root; *NsOSC3* has the highest expression level in the leaf; *NsOSC5* has the highest expression level in the stem and is almost not expressed in the root; and *NsOSC8* has the highest expression level in the stem, as well. Its expression in the transcriptome is consistent with its heat map (Figure 4).

### 3.5. Heterologous Expression of NsOSCs in Yeast

**Figure 5:**
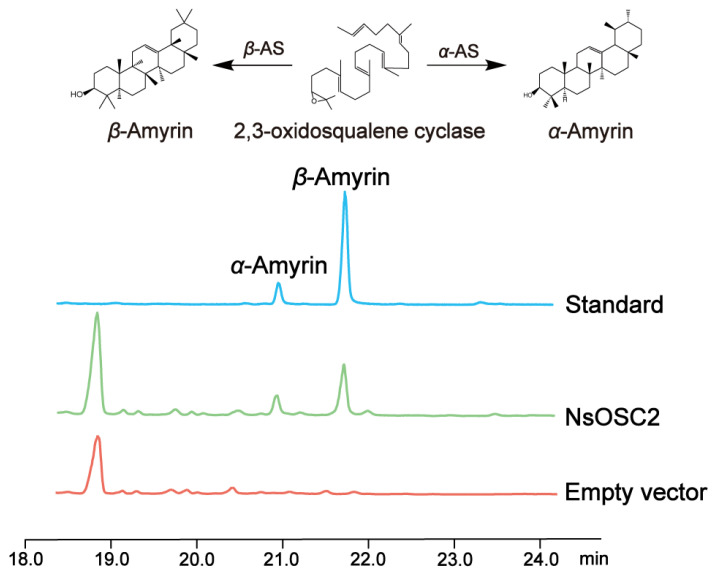
GC-MS analysis of the catalytic products produced by recombinant NsOSC2.

As a result of the above analysis, we found that oleanolic acid and ursolic acid have high connectivity and high content in medicinal parts. Additionally, both metabolites are derived from cinnamyl alcohol. Thus, we focus on the pathway that generates amyrin synthase from OSCs. Accordingly, NsOSC2 is clustered in the *β*-amyrin synthase and *α*-amyrin synthase genes; *NsOSC2* gene expression levels were highest in the gene cluster. NsOSC2 was therefore selected as the target gene for functional characterization. NsOSC2 was validated using heterologous yeast expression. The sequence was cloned from *N. scrophulariiflora* plants and connected to the yeast vector pYES2, and then the recombinant plasmid was transferred into mutant yeast GIL77. In addition, an empty pYES2 vector was used as a negative control. Yeast extracts expressing NsOSC2 were analyzed by GC-MS/MS, and the extracts were compared to the extracts using a mixture of *β*-amyrin synthase and *α*-amyrin synthase specimens (Supplementary Figure S11). NsOSC2 encodes a protein that catalyzes the conversion of 2,3-oxidosqualene to *β*-amyrin synthase and *α*-amyrin synthase (Figure 5).

### 3.6. Establishment of Regeneration System

**Figure 6:**
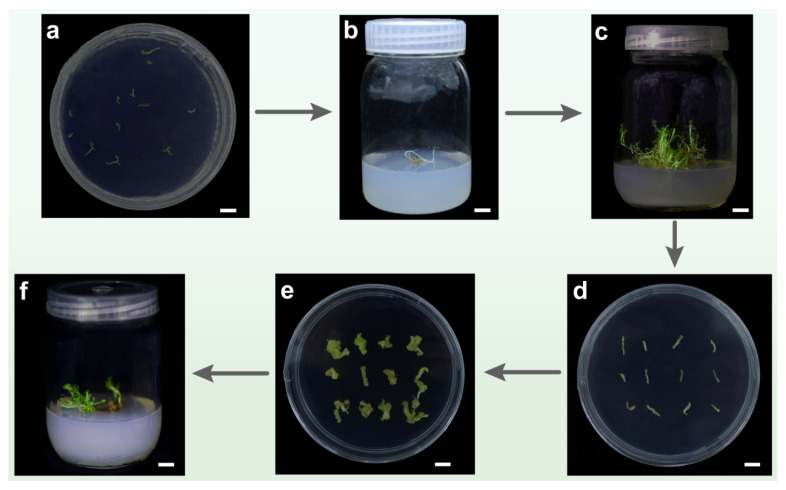
Establishment of regeneration system of *N. scrophulariiflora.* (**a**) Seed germination. (**b**) Growth and development of buds were transferred to subculture medium for 14 days. (**c**) After 45 days, *N. scrophulariiflora* has rooted and developed into a complete plant. (**d**) Cultivation of stem segments of *N. scrophulariiflora* as explants. (**e**) Healing induction and buds differentiation after 10 days of incubation on the healing medium. (**f**) The differentiated buds were transferred to a rooting medium, where they developed into complete plants. Bar = 1 cm.

A plant’s regeneration system is the basis for the implementation of plant genetic engineering, cell engineering, and other biotechnology, and it plays a key supporting role in plant-variety improvement, rare plant propagation, and plant physiological and biochemical research [31]. To establish an efficient *N. scrophulariiflora* regeneration system, we first selected full *N. scrophulariiflora* seeds to inoculate on seedling medium; inoculated 20–30 seeds in each petri dish; and cultured at 25 °C, 16 h light/8 h dark (Figure 6a). Seeds mature into buds after germination and sprouting, and after 14 d, the buds continue to elongate and become seedlings (Figure 6b). The seedlings were then transplanted into the rooting medium. Approximately 45 days later, *N. scrophulariiflora* seedlings grew into complete plants, resulting in sterile seedlings (Figure 6c). From this sterile seedling, a segment of the stem (about 1 cm) of *N. scrophulariiflora* was selected as the explant (Figure 6d). Callus differentiation was successfully induced after 10 d (Figure 6e), with the callus differentiation rate of 100% and the bud differentiation rate of 33% (Supplementary Table S8). For 30 d, the differentiated calli was transferred to the rooting medium to develop into complete plant (Figure 6f).

These results indicated that this study has successfully established a regeneration system for *N. scrophulariiflora*.

### 3.7. Establishment of Genetic Transformation System

**Figure 7:**
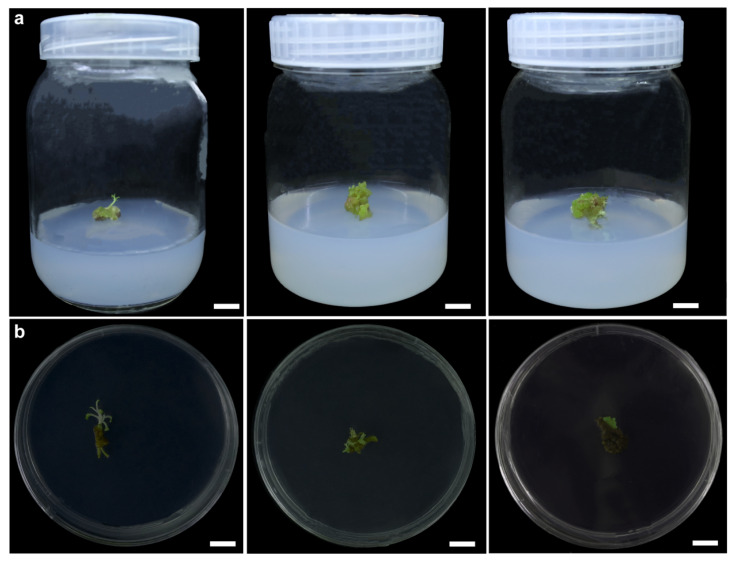
The process of genetic transformation of *N. scrophulariiflora.* (**a**) Healing tissues in good growth condition before transformation. (**b**) After 10 days of transformation, the *N. scrophulariiflora* healing tissue had differentiated into new green buds. Bar = 1 cm.

To establish the genetic transformation system for *N. scrophulariiflora,* we selected healthy calli to be soaked in GV3101/pCAMBIA1300*-OE-NsOSC2*. After being cocultured on acetosyringone-supplemented subculture medium for 2 d, the calli was transferred to kanamycin-supplemented medium (Figure 7a). New adventitious buds appeared in the callus 10 d after inoculation (Figure 7b).

The transgenic material turned brown when compared with the wild type (WT). A week later, new green buds emerged from their mother callus, displaying buds differentiation and normal morphology. The GFP-specific bands were not detected in the WT calli but were detected in the transgenic calli (Supplementary Figure S12). We then used RT-qPCR to detect the relative expression level of target gene in the transformed calli of *N. scrophulariiflor*. The results showed that *NsOSC2* expression level in six selected transformed calli of *N. scrophulariiflora* were higher than in WT (Supplementary Figure S13). Positive calli were detected (100%), and transformation efficiency was approximately 50%. These results indicated that the genetic transformation system was successfully established in *N. scrophulariiflora*.

## 4. Discussion

*Plantago asiatica,* the representative medicinal plant of the Plantaginaceae family, has been extensively studied, with over 60 chemical compounds isolated and identified [33], including polysaccharides, flavonoids and their glycosides, iridoids, triterpenes and sterols, trace elements, and volatile oils [34]. In this study, we conducted a comprehensive metabolite analysis of *N. scrophulariiflora* across its four major tissues: flowers, roots, stems, and leaves. We identified key compounds, such as flavonoids, sugars and their derivatives, terpenes, and alkaloids and their derivatives, which are consistent with those identified in *P. asiatica*. This metabolic overlap prospective outcomes from the combined effects of genetic conservation, ecological pressure, and natural selection within the Plantaginaceae family. These verdicts not only provide a theoretical basis for understanding the functional material basis of medicinal plants in this family, but also establish a foundation for further research into the biosynthesis of compounds in the Plantaginaceae family.

The rhizomes of *N. scrophulariiflora* contain numerous pharmacologically active compounds, chiefly triterpenoids, which exhibit a wide range of biological activities and demonstrate medicinal efficacy against various diseases. For example, ginsenosides are the main active ingredients in *P. ginseng* and belong to the triterpenoid class. They have various pharmacological effects, such as anti-tumor, anti-inflammatory, and antioxidant [35]. The triterpenoids in *C. asiatica* exhibit significant anti-inflammatory, wound healing, and skin protective effects [36]. Similarly, the triterpenoids in *Panax notoginseng* have demonstrated therapeutic efficacy in promoting blood circulation, removing blood stasis, reducing swelling, and relieving pain [37]. Meanwhile, triterpenoids from *Tripterygium wilfordii* have shown significant potential in treating the autoimmune diseases [38]. Moreover, the triterpenoids from *Ganoderma lucidum* are known as the “soul”; they exhibit important biological activities such as enhancing immunity and anti-tumor effects [39]. Our study provides the first comprehensive analysis of triterpenoids in *N. scrophulariiflora*, addressing a significant research gap. In addition, a comprehensive comparison of compounds between different tissues revealed the roots as the primary accumulation site for terpenes, with triterpenoids being the foremost subclass, encouraging attentive study of roots triterpenoid biosynthesis pathways. Therefore, further research was conducted on the synthesis of triterpenoids in the roots of *N. scrophulariiflora*. This study successfully identified 22 triterpenoids, which are mainly divided into two categories: pentacyclic triterpenoids and tetracyclic triterpenoids. This is consistent with the triterpenoids previously extracted from *P. asiatica* [40]. It is worth noting that tetracyclic triterpenoids exhibit certain differences: cucurbitacins were identified as the major tetracyclic triterpenoids in *N. scrophulariiflora*, but they were absent in *P. asiatica* [41]. On the other hand, pentacyclic triterpenoids showed remarkable consistency between the two species. The main pentacyclic triterpenoids in *N. scrophulariiflora*, including ursolic acid and oleanolic acid, were identical to those previously identified in *P. asiatica* [42]. This divergence highlights how the genetic factors determine metabolic pathways within associated taxa, as sometimes similar chemical substances can also exist in families and genera that are not morphologically similar. Remarkably, our results explained that the common existence of pentacyclic triterpenoids (e.g., ursolic acid) between these two morphologically disparate species proposes conserved biosynthetic mechanisms, possibly reflecting their phylogenetic kinship within the Plantaginaceae family.

The abundant triterpenoids in *N. scrophulariiflora* are derived from the mevalonic acid metabolic pathway, which synthesizes isoprene pyrophosphate and dimethylallyl pyrophosphate from acetyl-CoA. This metabolic pathway exists in all higher eukaryotes and many viruses [43]. This pathway is the starting point for the synthesis of various important natural products, including various bioactive substances, such as steroids and squalene. More than 100 triterpenoid skeletons with good biological activity have been discovered in plants, and under the catalysis of monooxygenase SE, squalene is further transformed into the precursor 2,3-oxidized squalene of many triterpenoid compounds [44]. These triterpenoid skeletons are further converted into different triterpenoids by cytochrome P450 monooxygenase, and, ultimately, these triterpenoids undergo glycosylation modification to form triterpenoid compounds [45].

We found that *NsOSC2* is a key gene in the synthesis of triterpenoids in *N. scrophulariiflora*, and it has been revealed to be a multifunctional aromatic resin alcohol synthase that can catalyze the formation of *α*-amyrin and *β*-amyrin from triterpenoid skeletons. The synthesis pathway of *α*-amyrin in plants has long been elucidated, but the current database is limited [46]. Most known amyrin synthases are specific synthases for producing *β*-amyrin [47,48], while *α*-amyrin synthases are all multifunctional enzymes that can catalyze the production of two or more products using OSC as a substrate, including pentacyclic triterpenoids such as *α*-amyrin, *β*-amyrin, and lupinol [49,50]. These two types of amyrin, as important medicinal ingredients, occupy a core position in the medicinal value of *N. scrophulariiflora*. These findings not only elucidate key genes involved in the triterpenoid biosynthesis in *N. scrophulariiflora* but also provide a theoretical basis for producing key triterpenoids through synthetic biology approaches.

From the perspective of resource conservation, this study found that the content of metabolites in roots and stem is not as high as that in flower and leaf. Therefore, during the collection process, the roots and stem can be preserved, both of which are beneficial for the propagation of *N. scrophulariiflora*. Moreover, terpenoid compounds are highly expressed in roots, leading to the speculation that terpenoid substances are mainly biosynthesized in roots, with some terpenoid compounds being transferred to different tissues. However, the specific mechanisms and substrate specificity require further research. This study successfully established an efficient regeneration and genetic transformation system for *N. scrophulariiflora*. By optimizing regeneration conditions and genetic transformation parameters, we achieved efficient regeneration from callus to whole plants. In addition, we also successfully established an efficient genetic transformation system.

Overall, this study not only reveals the key genek in the triterpenoid biosynthesis in *N. scrophulariiflora* but also provides a theoretical basis for producing key triterpenoids via synthetic biology approaches.

## 5. Conclusions

Based on extensive metabolomic and transcriptomic analyses of triterpenoid biosynthesis in *N. scrophulariiflora*, we found nine pentacyclic triterpenoid metabolites to be predominantly enriched in roots. Furthermore, we characterized eight key *NsOSC* genes, including *NsOSC2*, which was experimentally validated as a dual-functional enzyme capable of catalyzing the conversion of oxidosqualene into both *α*-amyrin and *β*-amyrin triterpenoid skeletons.

Additionally, we successfully established an efficient regeneration and genetic transformation system for *N. scrophulariiflora*, providing a crucial technical platform for functional genomics studies. These findings reveal the molecular mechanisms underlying triterpenoid synthesis in *N. scrophulariiflora* and lay the foundation for synthetic biology approaches aimed at producing key triterpenoids. This study not only deepens our understanding of triterpenoid biosynthesis in *N. scrophulariiflora* but also offers valuable insights for future molecular breeding.

## Data Availability

All original transcriptome data were submitted to the National Center for Biological Information (CNCB https://www.cncb.ac.cn/), China, with the BioProject accession (PRJCA027400).

## Supplementary Materials

The following supporting information can be downloaded at https://www.mdpi.com/article/10.3390/plants14101562/s1.
