# Supplementary material for: Multi-Omics and Functional Insights into Triterpenoid Biosynthesis Pathways in Neopicrorhiza scrophulariiflora (Pennell) D.Y.Hong
Source: Plants (Basel). 2025 May 21;14(10):1562. doi: 10.3390/plants14101562 (PMC12114848; doi:10.3390/plants14101562)
Supplement: Supplementary file 1 [file plants-14-01562-s001.zip › Supplementary Figures.pdf]

Supplementary Figures

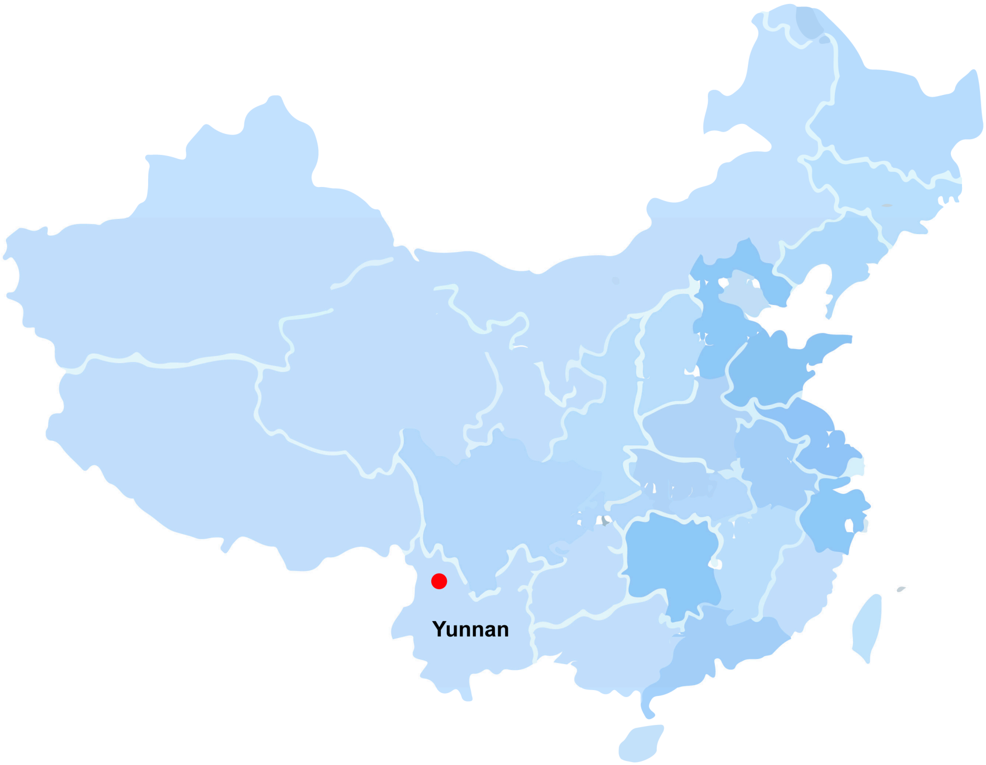

Figure S1 Map of main producing areas of *N. scrophulariiflora*. Red dot represents the source of research materials.

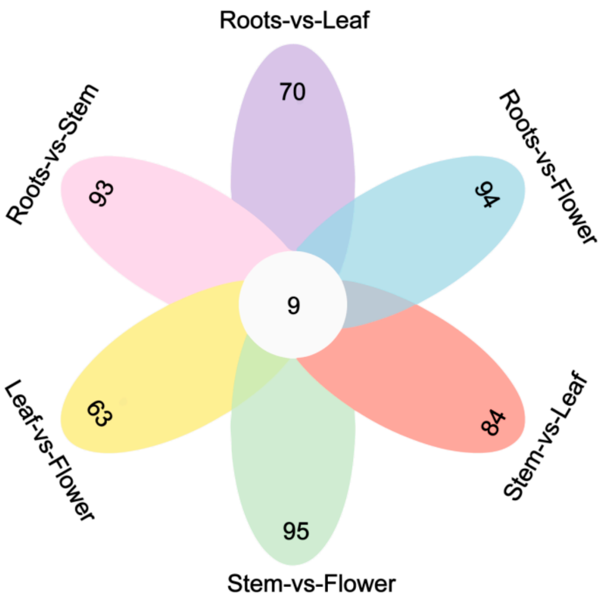

Figure S2 Venn diagram for DAMs between different tissues of roots, stem, leaf and flower.

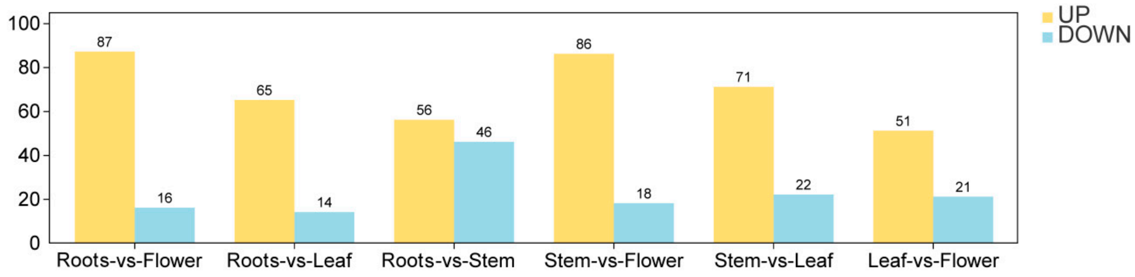

Figure S3 Comparison of differentially accumulated metabolites (DAMs) in different tissues. Yellow represents up-accumulated metabolites, while blue represents down-accumulated metabolites.

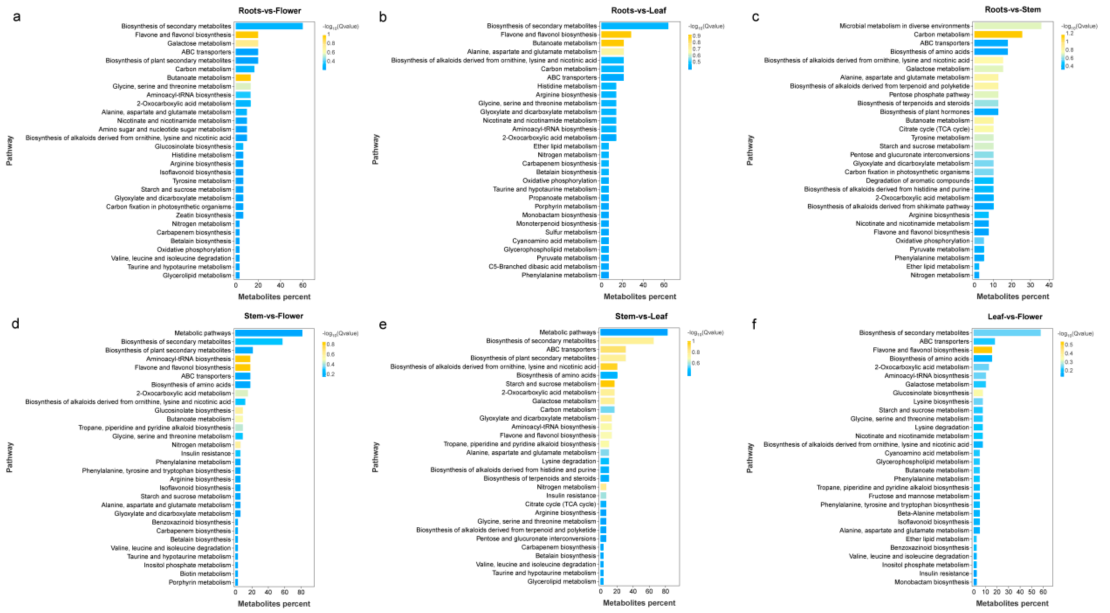

Figure S4 DAMs enrichment analysis in metabolome. The vertical axis shows the term name, and the horizontal axis shows the enriched factor.

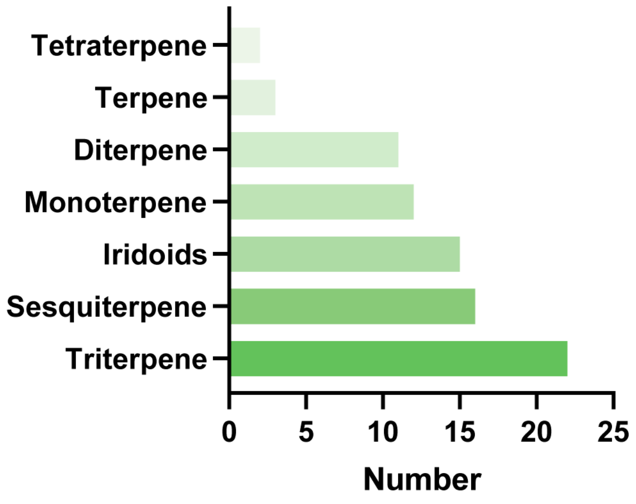

Figure S5 Number of terpenes. The vertical axis displays categories of terpenes., and the horizontal axis displays their quantities. The column length represents the number of DAMs.

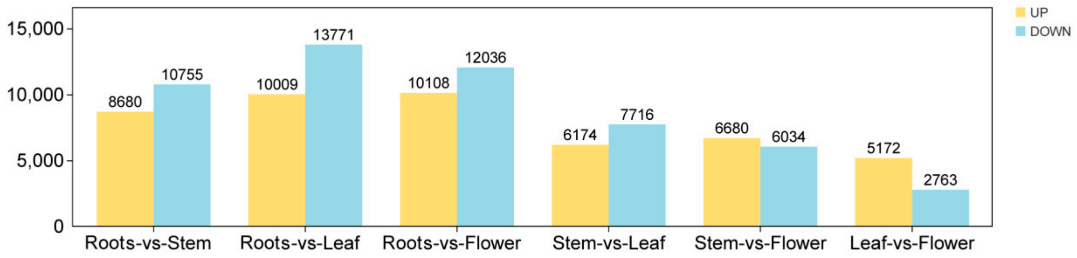

Figure S6 Comparison of differentially expressed genes (DEGs) in different tissues. Yellow represents upregulated genes, while blue represents downregulated genes.

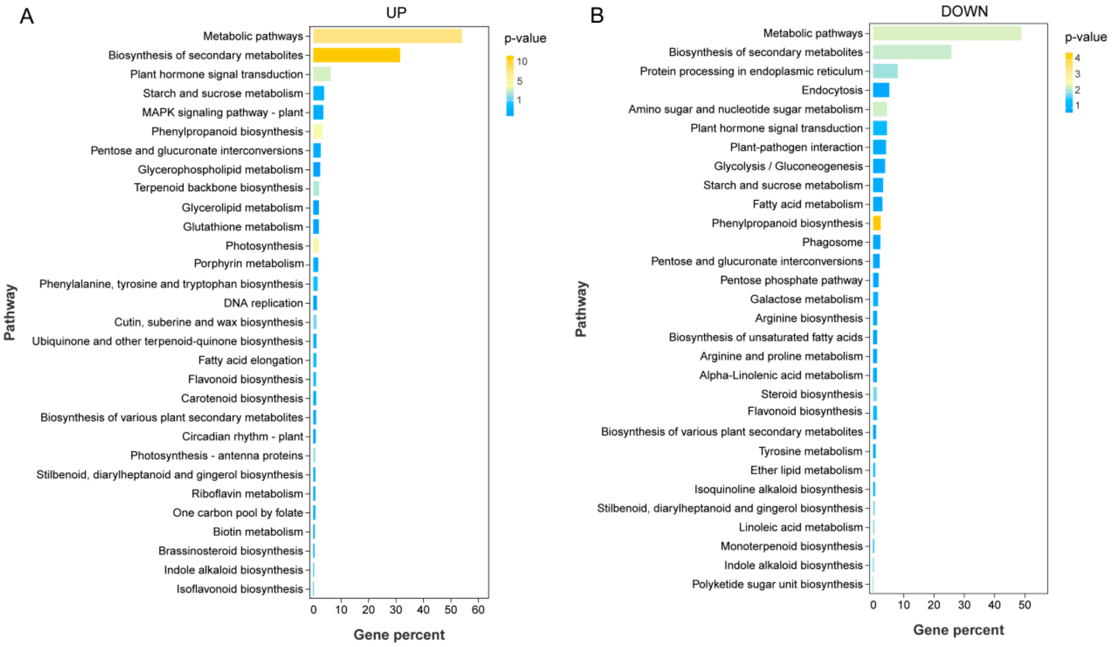

Figure S7 Enrichment analysis of differentially expressed genes in the transcriptome. a. The KEGG enrichment analysis of co-upregulated DEGs. b. and co-downregulated DEGs of different tissues. The vertical axis indicates the term name, and the horizontal axis indicates the enriched factor.

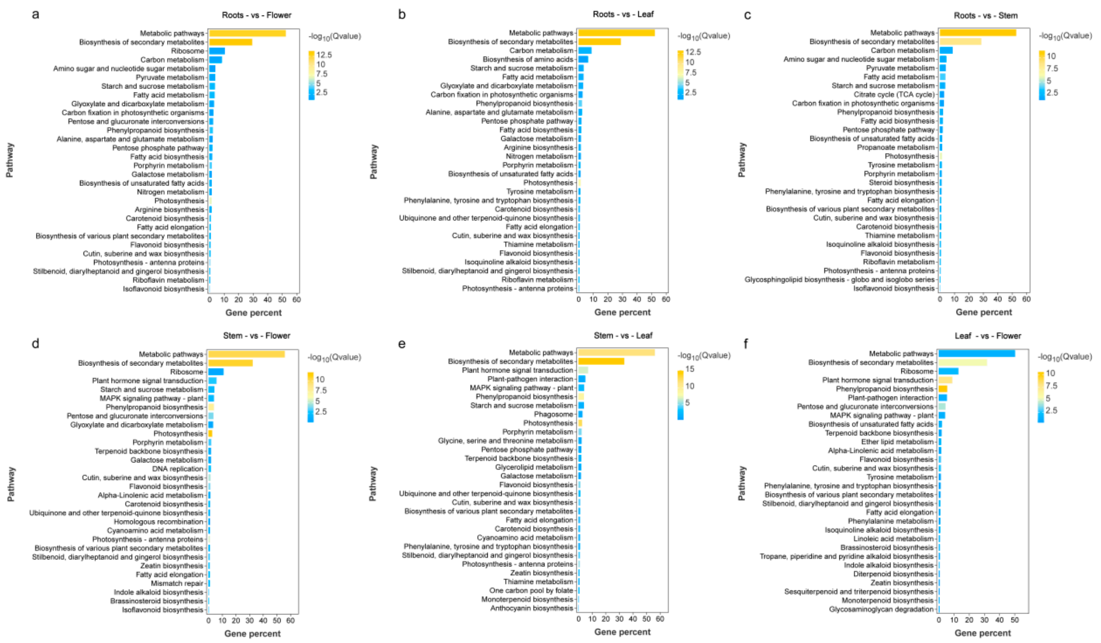

Figure S8 KEGG pathway analysis of DEGs. The vertical axis indicates the term name, and the horizontal axis indicates the enriched factor.

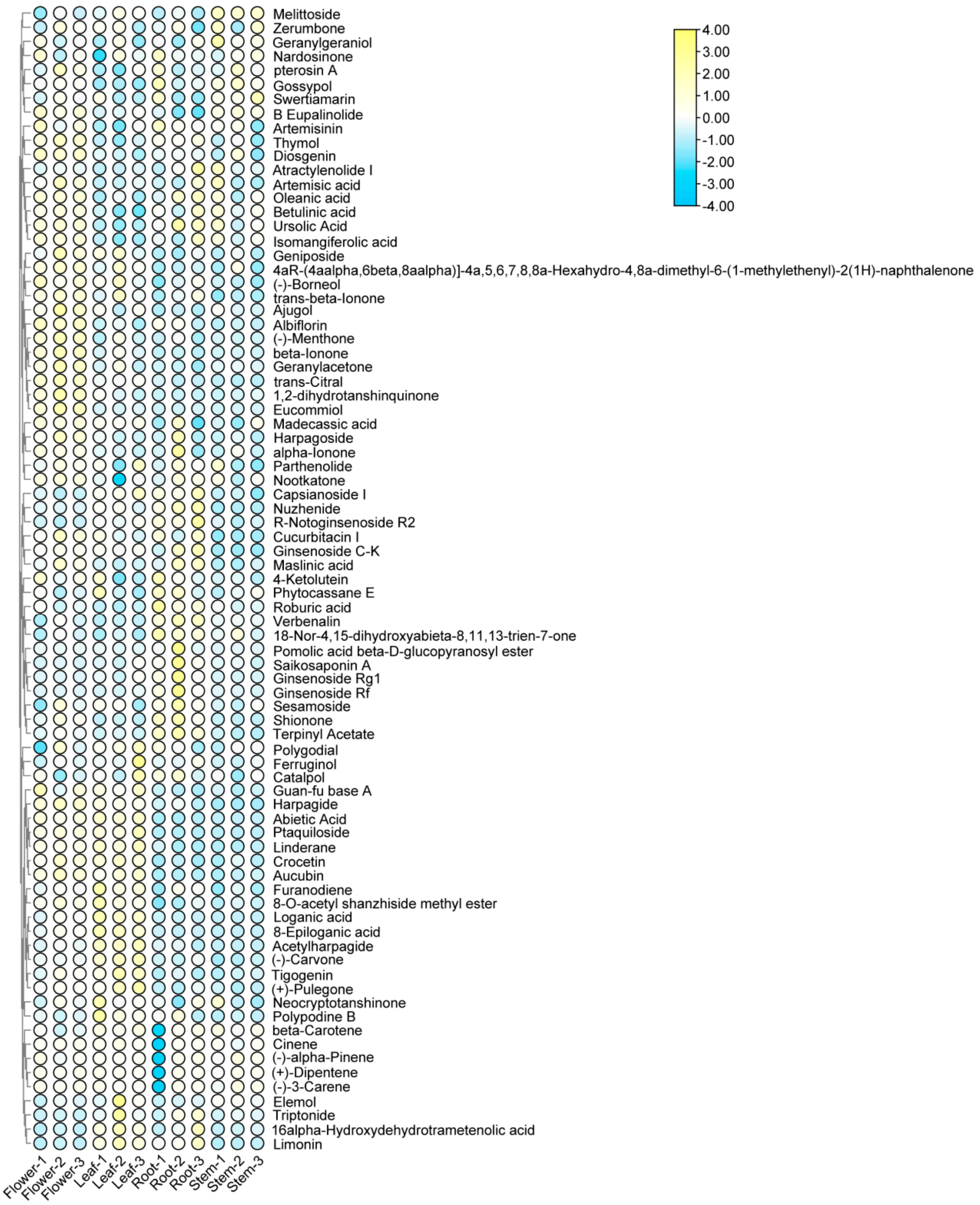

Figure S9 Heatmap analysis of terpenoids content from flower, leaf, stem and roots in *N. scrophulariiflora*. The heat map displays the *Z*-score calculated from the relative content of metabolites in different tissues and indicated from low (blue) to high (yellow).

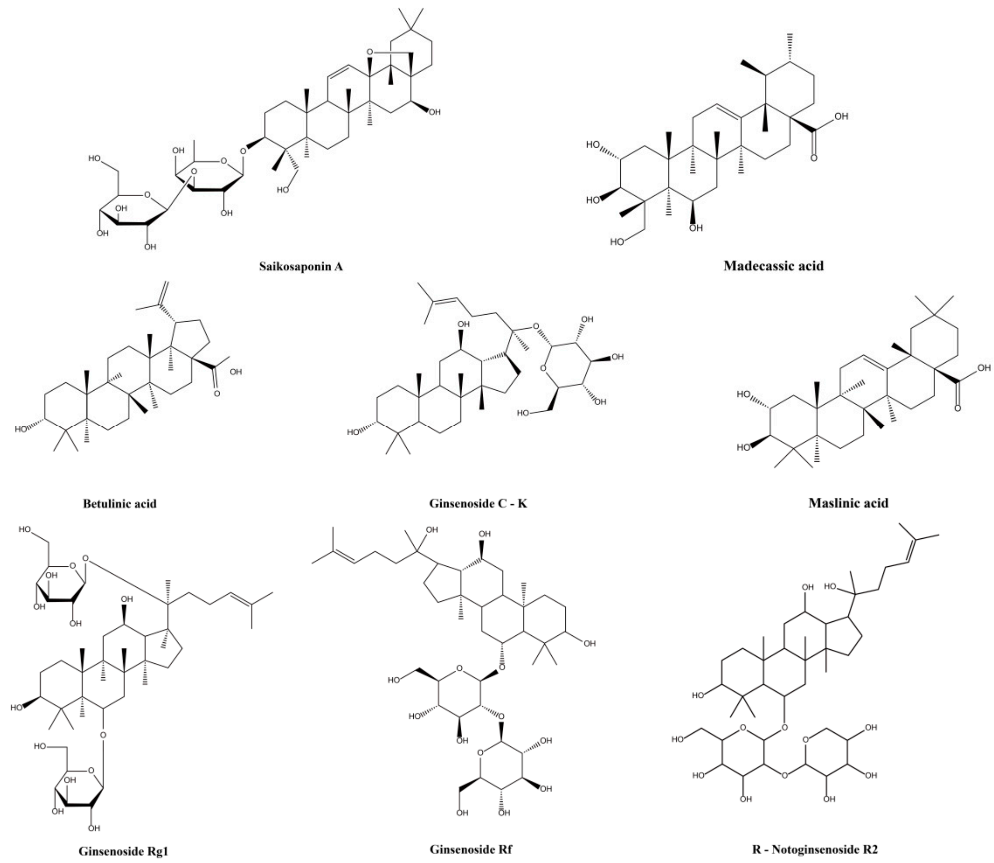

Figure S10 The structural formula of triterpenoids, including saikosaponin A, madecassic acid, betulinic acid, ginsenoside C-K, maslinic acid, ginsenoside Rg1, ginsenoside Rf, R-notoginsenoside R2.

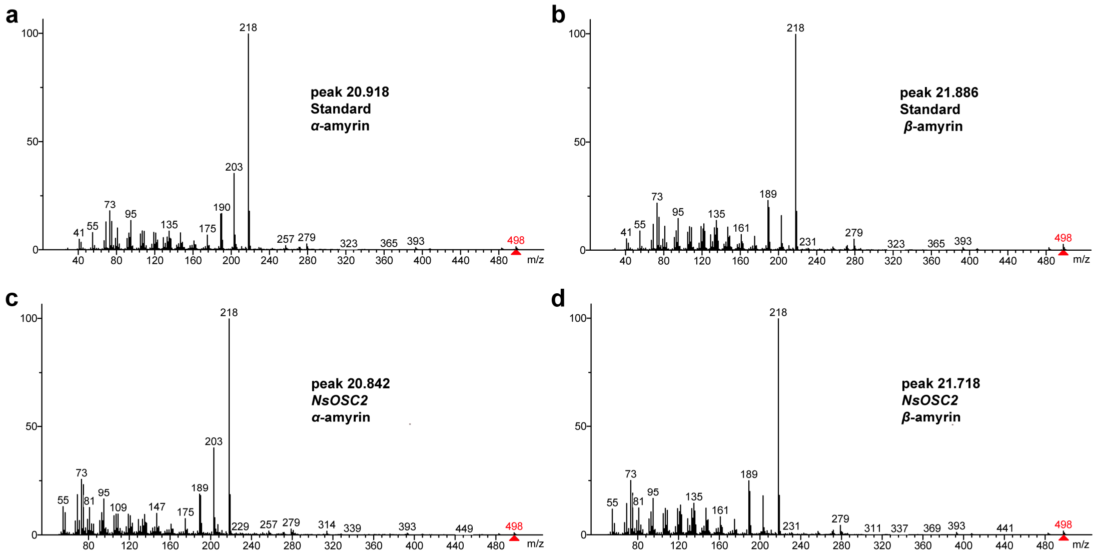

Figure S11 Mass spectra identification for compounds. a. The molecular weight and peak time of α-amyrin standard; b. The molecular weight and peak time of β-amyrin standard; c and d. The molecular weight and peak time of the sample.

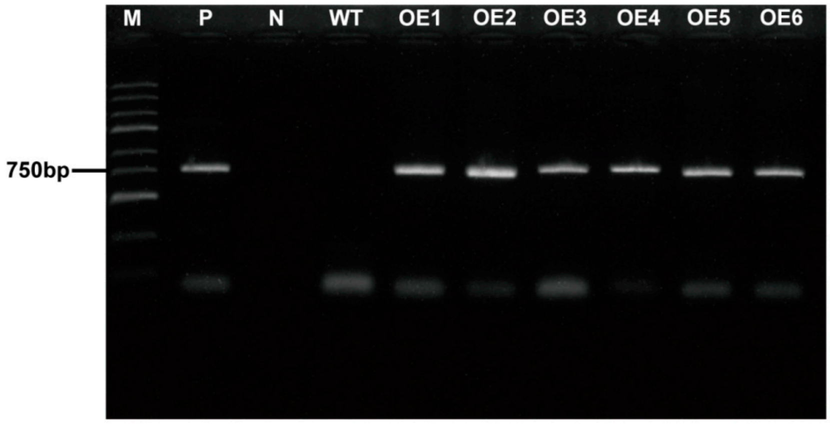

Figure S12 PCR confirmation of transgenic plants. P represents positive control. N represents negative control using double-distilled water.

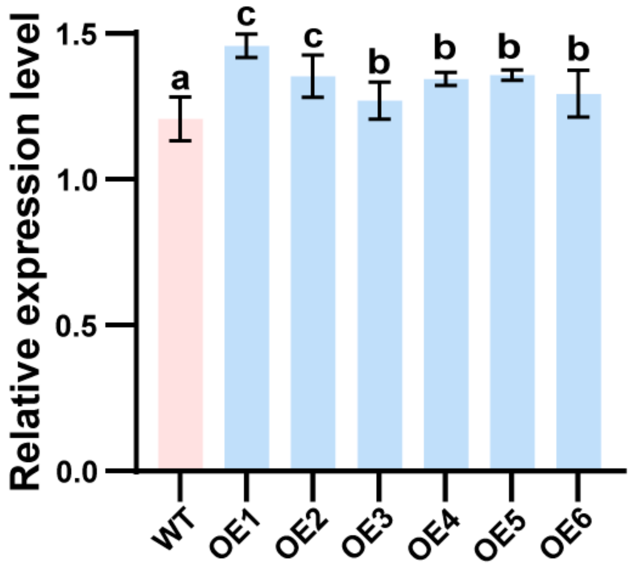

Figure S13 Relative expression level of *NsOSC2* in WT and OE-*NsOSC2* (OE1-6). WT: wild-type *N. scrophulariiflora* plant, OE1-6: transgenic lines OE1-6.
