# Supplementary material for: Multi-Omics and Functional Insights into Triterpenoid Biosynthesis Pathways in Neopicrorhiza scrophulariiflora (Pennell) D.Y.Hong
Source: Plants (Basel). 2025 May 21;14(10):1562. doi: 10.3390/plants14101562 (PMC12114848; doi:10.3390/plants14101562)
Supplement: Supplementary file 1 [file plants-14-01562-s001.zip › Supplementary Tables.pdf]

Supplementary Table S1 The triterpenoid information

NO	Name	Structure	Rf	Function
1	ginsenoside	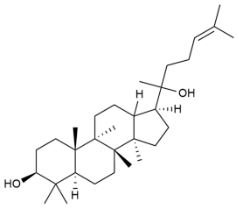	Liu, X., Xiang, J., Fan, S., Chen, X., Peng, C., & Xu, Z. (2025). 20S-Ginsenoside Rh2, the major bioactive saponin in *Panax notoginseng* flowers, ameliorates cough by inhibition of NaV1.7 and TRPV1 channel currents and downregulation of TRPV1 expression. Journal of ethnopharmacology, 336, 118716. https://doi.org/10.1016/j.jep.2024.118716	preventing cardiovascular diseases
2	glycyrrhizic acid	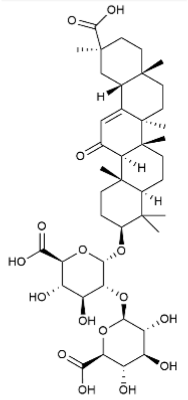	Peng, D., Wang, A., Shi, W., & Lin, L. (2024). Pentacyclic triterpenes, potential novel therapeutic approaches for cardiovascular diseases. Archives of pharmacal research, 47(8-9), 709–735. https://doi.org/10.1007/s12272-024-01510-4	anti-inflammatory, antioxidant, antifibrotic immunomodulatory effects
3	loganin	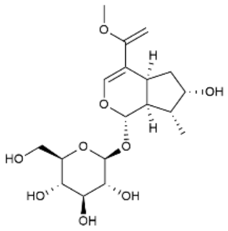	Xie, Y., Qin, X., Zhou, T., Zhou, Y., Tang, L., Wang, J., Lin, Z., Dong, Q., & Sun, P. (2024). Investigating the protective effect of loganin in ovariectomy-induced bone loss through network pharmacology and molecular docking. Experimental and therapeutic medicine, 28(5), 417. https://doi.org/10.3892/etm.2024.12706	anti-inflammatory, memory-enhancing

4	saikosaponin	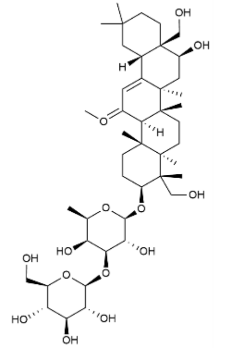	Yang, T., Li, X., Wang, X., Meng, X., Zhang, Z., Zhao, M., & Su, R. (2024). Combination of histological and metabolomic assessments to evaluate the potential pharmacological efficacy of saikosaponin D. Journal of pharmaceutical and biomedical analysis, 242, 116001. https://doi.org/10.1016/j.jpba.2024.116001	
5	*β*-amyrin	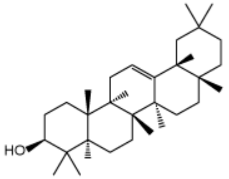	Luz D. M. Gómez-Pulido,Rafael C. González-Cano,José J. Benítez,Ernesto Redondo Domínguez, & Antonio Heredia (2022). Structural analysis of mixed *α*- and *β*-amyrin samples. Royal Society Open Science, 9 (4), 0-0. https://doi.org/10.1098/rsos.211787	sedative, anti-inflammatory, antibacterial, liver-protecting, nephritis-resisting, immunity-regulating
6	*α*-amyrin	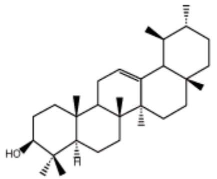		
7	lupeol	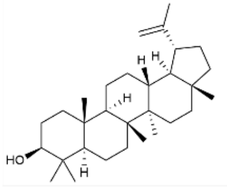	Sen, K., Kumar Das, S., Ghosh, N., Sinha, K., & Sil, P. C. (2024). Lupeol: A dietary and medicinal triterpene with therapeutic potential. Biochemical pharmacology, 229, 116545. https://doi.org/10.1016/j.bcp.2024.116545	antioxidant, anti-inflammatory, skin healing promoting

8	taraxerol	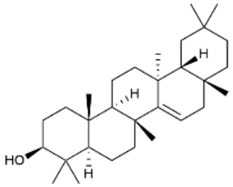	Ragasa, C.Y, & Cornelio, K.B (2013). Triterpenes from Euphorbia hirta and their cytotoxicity. Chinese journal of natural medicines, 11 (5), 528-33. https://doi.org/10.1016/S1875-5364(13)60096-5	anti-tumor
9	oleanolic acid	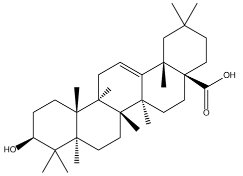	Jeevitha, C. M., Ravichandiran, K., Tanuja, T., & Parani, M. (2025). Transcriptome sequencing and identification of full-length genes involved in the biosynthesis of anticancer compounds Oleanolic acid and Ursolic acid in *Achyranthes aspera* L. Gene, 933, 148964. https://doi.org/10.1016/j.gene.2024.148964	antiviral, anti-HIV, antibacterial, antifungal, anticarcinogenic, anti-inflammatory, hepatoprotective, gastroprotective, hypolipidemic anti-atherosclerotic activities
10	ursolic acid	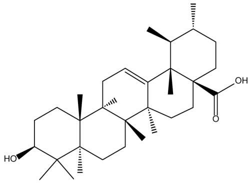		antioxidant, anti-inflammatory, antidiabetic effects

Supplementary Table S5 PCR primer sequences

Primer names	Sequence (5'-3')
NsOSC2-F	AGCTTGGTACCGAGCTCGGAATGTGGAAGCTCAAAATTGCTG
NsOSC2-R	GCAATTTTGAGCTTCCACATTTGGGAGGGCCAGACAC
NsOSC3-F	AGCTTGGTACCGAGCTCGGAATGTGGAAGCTGAAAATTGCAGAA
NsOSC3-R	GCAATTTTGAGCTTCCACATCATTACTTTCAGTCCCTCTGCTG
NsOSC5-F	AGCTTGGTACCGAGCTCGGAATGTGGAGGCTTAAACTATCTGAAG
NsOSC5-R	TGGCGGCCGTTACTAGTGGACTGAAGTGATAAAACCCAATCAACTG
NsOSC8-F	TTGGTACCGAGCTCGGATCCATGGAGTCTACTCTCAAGAAATATTTCGG
NsOSC8-R	CACTGGCGGCCGTTACTAGTCATGATTTTATAAACATCGTTCTTCC
NsOSC2-P1300-F	GACGAGCTCGGTACCCGGGGAATGTGGAAGCTCAAAATTGCTGA
**NsOSC2-P1300-**NsOSC2-P1300-R	TCACCATGTCGACTCTAGAGGATTGGGAGGGCCAGACAC

Supplementary Table S6 Quantitative Analysis of identified metabolites

Mode	ALL Metabolites	KEGG
POS	732	214
NEG	550	247
Total	1282	461

Note: “Mode” indicates that the mode of the MS analysis was mainly divided into a positive ion mode (POS) and a negativeionmode(NEG). “Allmetabolites” indicates the substances extracted by UPLCMS/MS; “MS1KEGG”indicates the metabolites that were annotated to the KEGG pathway.

Supplementary Table S7 RT-qPCR primer sequences

Primer names	Sequence (5'-3')
NsOSC2-F-Y	CTGGTGGTTGTAGATGTAGCGA
NsOSC2-R-Y	GTGACGTATGAGAAGGCCACTA
NsOSC3-F-Y	CAGAATAGAGATGGTGGATGGGG
NsOSC3-R-Y	CGTGCTTTGTTCCAATCGACTT
NsOSC5-F-Y	GGAGATGGAGCGATGGTCAAA
NsOSC5-R-Y	ATTCGCCCTGGATGTATTGGAA
NsOSC8-F-Y	CCTGTTCAACATCCTTGACGGT
NsOSC8-R-Y	CGGACTAATGCTTTTCTGGATGAG

Supplementary Table S8 Differentiation of stem explants into callus

Number of stem explants	Number of callus differentiation	Number of bud differentiation
12	12	4
